# Role of SARS-CoV-2 in Modifying Neurodegenerative Processes in Parkinson’s Disease: A Narrative Review

**DOI:** 10.3390/brainsci12050536

**Published:** 2022-04-22

**Authors:** Jeremy M. Morowitz, Kaylyn B. Pogson, Daniel A. Roque, Frank C. Church

**Affiliations:** 1 Developmental and Stem Cell Biology Program, Duke University, Durham, NC 27708, USA; jeremy.morowitz@duke.edu; 2 School of Medicine, The University of North Carolina at Chapel Hill, Chapel Hill, NC 27599, USA; kaylyn_pogson@med.unc.edu; 3 Department of Neurology, The University of North Carolina at Chapel Hill School of Medicine, Chapel Hill, NC 27599, USA; droque@neurology.unc.edu; 4 Department of Pathology and Laboratory Medicine, The University of North Carolina at Chapel Hill School of Medicine, Chapel Hill, NC 27599, USA

**Keywords:** Parkinson’s disease, COVID-19, SARS-CoV-2, neurodegenerative disorder, older adults, brain, alpha-synuclein, angiotensin converting enzyme 2 receptor, neuroinflammation

## Abstract

The COVID-19 pandemic, caused by SARS-CoV-2, continues to impact global health regarding both morbidity and mortality. Although SARS-CoV-2 primarily causes acute respiratory distress syndrome (ARDS), the virus interacts with and influences other organs and tissues, including blood vessel endothelium, heart, gastrointestinal tract, and brain. We are learning much about the pathophysiology of SARS-CoV-2 infection; however, we are just beginning to study and understand the long-term and chronic health consequences. Since the pandemic’s beginning in late 2019, older adults, those with pre-existing illnesses, or both, have an increased risk of contracting COVID-19 and developing severe COVID-19. Furthermore, older adults are also more likely to develop the neurodegenerative disorder Parkinson’s disease (PD), with advanced age as the most significant risk factor. Thus, does SARS-CoV-2 potentially influence, promote, or accelerate the development of PD in older adults? Our initial focus was aimed at understanding SARS-CoV-2 pathophysiology and the connection to neurodegenerative disorders. We then completed a literature review to assess the relationship between PD and COVID-19. We described potential molecular and cellular pathways that indicate dopaminergic neurons are susceptible, both directly and indirectly, to SARS-CoV-2 infection. We concluded that under certain pathological circumstances, in vulnerable persons-with-Parkinson’s disease (PwP), SARS-CoV-2 acts as a neurodegenerative enhancer to potentially support the development or progression of PD and its related motor and non-motor symptoms.

## 1. Introduction

### 1.1. COVID-19 Pandemic

In late 2019, a novel coronavirus appeared in Wuhan, China, which was given the name severe acute respiratory syndrome coronavirus 2 (SARS-CoV-2), after similarities to the SARS virus were noted [1,2,3]. The resulting disease caused by SARS-CoV-2 was termed coronavirus disease 2019 (COVID-19) [1,2]. SARS-CoV-2 is a beta coronavirus composed of a single-stranded RNA virus thought to have originated in bats. SARS-CoV-2 binds to the angiotensin-converting enzyme 2 (ACE2) receptor on type I and type II pneumocytes [1,2]. Because of what cells the virus binds to, COVID-19 infection usually includes fever, cough, and dyspnea, with progression to pneumonia or sepsis in more critical cases (see Refs. [4,5,6] and references cited therein). In addition, as described by Chen et al. [4] and Jiang et al. [5], severe COVID-19 cases lead to below-normal oxygen levels in the blood (hypoxemia) and may require breathing assistance through mechanical ventilation. Furthermore, as the SARS-CoV-2 infection evolves in some individuals, a potent pro-inflammatory state can lead to acute respiratory distress syndrome (ARDS) and cytokine storm syndrome (CSS); these two conditions have caused much of the morbidity and mortality found globally due to SARS-CoV-2 [1,2,3,4,5,6]. Also contributing to a poorer outcome from COVID-19 infection are certain risk factors and preexisting illnesses, such as older age, male sex, and metabolic disorders, including obesity and diabetes [7].

Globally, as of 22 February 2022, there have been 423,437,674 confirmed cases of COVID-19, including 5,887,328 deaths, reported to WHO. As of 21 February 2022, a total of 10,407,355,583 vaccine doses have been administered. In the USA, as of 21 February 2022, there have been 77,729,484 confirmed cases of COVID-19 with 926,287 deaths, reported to WHO. As of 18 February 2022, a total of 534,803,540 vaccine doses have been administered [8].

### 1.2. Neurological Mayhem during SARS-CoV-2 Infection

SARS-CoV-2 primarily targets the respiratory tract, although the symptoms found are widely recognized to be very heterogeneous, ranging from minimal symptoms to significant hypoxia with ARDS [3]. Early in the COVID-19 pandemic, there was substantial evidence of neurological symptoms, including headache, dizziness, a residual cognitive cloud, and changes by the loss in taste and smell, as reported by Chen et al. [9], Russell et al. [10], Lahiri and Ardila [11], Yachou et al. [12], and Pezzini and Padovani [13]. The clinical evidence suggests that SARS-CoV-2 is the cause of the neurological symptoms in COVID-19 [9,10,11,12,13]. It has been shown previously that several human respiratory viruses, including coronaviruses, are either neuro-invasive or neurotropic, which can then promote neuropathological problems. The cytokine storm generated in response to SARS-CoV-2 [14,15] and the ensuing immune reaction are key components that support invasion of the central nervous system (CNS).

### 1.3. Parkinson’s Disease

Parkinson’s disease (PD) occurs when the dopaminergic neuronal cells in the pars compacta region of the substantia nigra have degenerated, leading to inadequate postsynaptic levels of the neurotransmitter dopamine, as presented in these Refs. [16,17,18]. Prototypical early symptoms in PD usually involve a gradual, years-long decline in previously-learned motor function, making the early diagnosis of PD challenging [18]. PD is traditionally described as a motor system disorder with bradykinesia (slowness of movement) and, at minimum, one of three other cardinal symptoms: rigidity (stiffness of the limbs and trunk), postural instability (impaired balance and coordination), and/or tremor (trembling in hands, arms, legs, and face) (see Refs. [19,20,21,22,23] and references cited therein). Importantly, non-motor symptoms are also experienced by many of those with PD, including but not limited to constipation, urinary dysfunction, depression, psychosis, apathy, and sleep disorders, as reviewed in Refs. [22,24,25,26,27].

**Figure 1:**
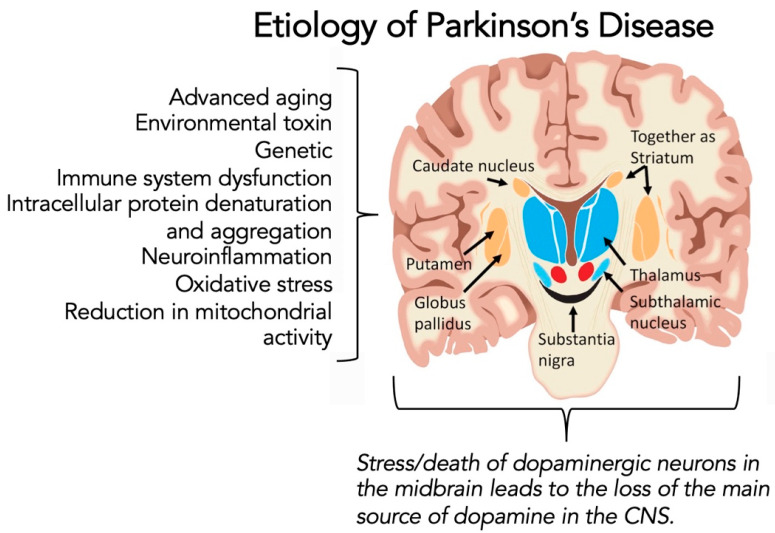
Etiology of Parkinson’s disease showing the major causes of the disorder that promote the loss of dopaminergic neurons in the substantia nigra pars compacta mid-brain region. Abbreviation used: CNS, central nervous system.

PD occurs most commonly in people aged over 60 years old [18]. In this older age group, most cases of PD occur sporadically and are due to a complex mixture of etiologies that may include neuroinflammation, oxidative stress, immune system dysfunction, reduction in mitochondrial activity, genetic mutation, disruption in intracellular protein denaturation and aggregation, and other environmental factors (Figure 1) [16,17,18,28,29]. Interestingly, cases of PD in younger people (age younger than 40 years old) are usually linked to particular genotypes [30]. At present, PD remains an incurable disease. As such, treatment goals in PD management center on symptomatic management, with only cardiovascular exercises [31,32] and therapies [27,33] known to impact disease progression. In addition, however, many persons-with-Parkinson’s disease (PwP) use complementary and alternative medicine (CAM) approaches, including over-the-counter supplements, mindfulness meditation, and several other forms of exercise to manage better their quality of life (for further information, please consult Refs. [23,34,35,36]).

Over one million people in the USA are living with PD, with ~60,000 new cases diagnosed nationally each year [28]. The global prevalence of PD is believed to be between 7–10 million people.

### 1.4. Potential Link between COVID-19 Infection and Parkinson’s Diseases

Our interest in this topic is intertwined by several common features [3,9,10,11,12,13,37,38,39,40,41,42,43,44,45], which include:

1. the older age of susceptibility for both severe COVID-19 infection and developing PD;
2. the intense pathophysiology of the pro-inflammatory response of the host to SARS-CoV-2;
3. the history of other viruses (including coronaviruses) invading the CNS and promoting neurodegenerative disorders;
4. the growing evidence that SARS-CoV-2 has a neurotropic potential;
5. and, the circumstantial reports in the literature of worsening of symptoms in persons previously diagnosed with PD.

Literature searches for this review article were performed with PubMed and Google Scholar with search terms in various combinations: “Parkinson’s disease”, “Parkinson disease”, “SARS-CoV-2”, “COVID-19”, “neurodegeneration”, “neuropathology”, “cerebrospinal fluid”, “inflammation”, “neuroinflammation”, “reactive oxygen species”, “pro-inflammatory cytokine”, “innate immunity”, “adaptive immunity”, “cytokine storm syndrome”, “oxidative stress”, “Coronavirus”, “alpha-synuclein”, “hyper-inflammation”, “motor symptoms”, “non-motor symptoms”, “ACE2 receptor”, “neurotropism”, “neuroinvasive”, “spike protein”, and dopaminergic neuron”. All articles were in English and appeared in peer-reviewed journals. The articles (primarily consisted of reviews, basic research articles, clinical case studies, and editorials were considered) were compiled by the authors, and then appropriately added to the journal article in their representative sections. Since the COVID-19 pandemic is recent, years searched were 2019–2022.

Thus, the goal for this Narrative Review was to understand the potential role of SARS-CoV-2 either to accelerate existing PD cases or to help initiate PD and related-parkinsonism disorders. We begin by discussing some of the critical features of SARS-CoV-2 and the pathophysiology of the infection.

## 2. COVID-19 Infection

### 2.1. Structure and Function of SARS-CoV-2

SARS-CoV-2 is an enveloped, single-stranded positive, 29,903 bp RNA coronavirus [46]. Coronaviruses can be divided into four genera based on their genomic structure: α, β, γ, and δ [47]. SARS viruses are classified as β coronaviruses and can be transmitted to and among human hosts through means of direct contact, droplet exposure, and airborne routes [47]. The virus penetrates host cells through endocytosis or membrane fusion [3]. Upon entrance into the cell, the viral contents are released into the host. Viral RNA localizes to the nucleus where it undergoes replication and produces mRNA which is utilized to synthesize viral proteins. New viral particles are created from the combination of four structural viral proteins; spike (S), membrane (M), envelope (E), and nucleocapsid (N) [48].

### 2.2. Role of the Spike Protein

The spike protein is crucial to pathophysiology of the virus and is comprised of a transmembrane trimetric glycoprotein protruding from the viral surface [3]. The spike protein is responsible for the diversity of coronaviruses and determines host tropism. The spike protein consists of two functional subunits: S1, which is responsible for binding to the host cell receptor, and S2, which is responsible for the fusion of the host cell and viral membranes [49]. Following the binding of SARS-CoV-2 to the host receptor, the spike protein undergoes protease cleavage [50]. Cleavage at the S2 site activates the spike for membrane fusion, via irreversible conformational changes [51]. The coronavirus spike is unusual among viruses because multiple distinct proteases, such as furin, transmembrane protease serine 2, and cathepsin L, have the capability to cleave and activate it.

### 2.3. Angiotensin-Converting Enzyme 2 (ACE2) Receptor

SARS-CoV-2 infects cells via the angiotensin-converting enzyme 2 (ACE2) receptor [52]. The ACE2 receptor is well-known for its role in the regulation of blood pressure, maintaining homeostasis through negatively regulating the renin-angiotensin system [53]. ACE2 receptors are endothelium-bound carboxypeptidases that are highly expressed in the endothelial cells of the arteries, arterioles, and venules of the heart and kidney [54]. Furthermore, recent single-cell RNA sequencing data found high expression of ACE2 receptors in pulmonary type II alveolar cells, respiratory epithelial cells, myocardial cells, ileum epithelial cells, and esophageal epithelial cells [55]. Interestingly, the expression profiles of ACE2 expression in the lung are seen to be age-dependent in mice [3], and circulating levels of ACE2 have measurable differences based on sex in humans [56]. Due to the variable expression, spatially, temporally, and individually, the ACE2 receptor and its interaction with SARS-CoV-2 plays a crucial role in understanding COVID-19.

### 2.4. Pathophysiology

Once the virus has invaded the body, common pathophysiologic mechanisms of COVID-19 include dysregulation of cytokines and chemokines, disruption of the innate immune response, infection of immune cells, viral cytopathic effects, and autoimmunity [57]. COVID-19 has several typical clinical manifestations, including fever, cough, sore throat, shortness of breath, diarrhea, and fatigue. Beyond this, there is mounting evidence that COVID-19 patients can experience neuropsychiatric manifestations as well, including anosmia, ageusia, stroke, acute inflammatory demyelinating polyneuropathy (AIDP), acute necrotizing hemorrhagic encephalopathy, toxic-metabolic encephalopathy, headache, myalgia, central respiratory failure, myelitis, and ataxia [58].

### 2.5. Cytokine Storm Syndrome

This myriad of disease manifestations makes it difficult to understand the exact mechanisms of illness. An area of interest within the clinical and research community has been the “cytokine storm”, a system-wide elevation of pro-inflammatory cytokines, which has been observed in cases of severe COVID-19 [59]. This cytokine storm profile resembles that of secondary hemophagocytic lymphohistiocytosis, a syndrome that leads to fulminant and fatal hypercytokinemia with multiorgan failure [60]. The cytokine storm is characterized by the presence of increased interleukins IL-2 and IL-7, granulocyte-colony stimulating factor, interferon-alpha, monocyte chemoattractant protein 1, macrophage inflammatory protein 1-alpha, and tumor necrosis factor-alpha [61]. This cytokine storm can cause acute respiratory distress syndrome, pneumonia, and even multiple organ failure [62]. From here we delve into the neurological links that exist for SARS-CoV-2, and a direct link between SARS-CoV-2 and proteins involved in the initiation process of PD.

## 3. COVID-19, Neural Entry, and the Functional Role in Neurodegeneration

### 3.1. Neurological Interactions of SARS-CoV-2

COVID-19′s impact upon neural tissues can occur through two mechanisms: direct invasion or indirect effects due to hyperinflammation [46]. Infected patients exhibit high concentrations of SARS-CoV-2 in the nares, which causes inflammation of the olfactory nerves and results in structural damage to odor receptors, contributing to anosmia [63]. Interestingly, a study from 2008 demonstrated the ability of SARS-CoV to induce neuronal cell death in mice via invasion of the brain through the nose and olfactory epithelium [64]. Neurotropism may similarly occur in humans via viremia or by crossing the cribriform plate of the ethmoid bone [63]. Additionally, SARS-CoV-2 could reach cerebral circulation via systemic circulation, breaching the blood-brain barrier through ACE2 receptors on the endothelial cells which line blood capillaries in the brain, potentially causing rupture and thrombus [65]. Once within the CNS, due to expression of ACE2 receptors throughout the brain, SARS-CoV-2 can infect nerve cells, including neurons within the medulla oblongata, which acts as an autonomic regulatory center for heart and lung function [66]. It has been speculated that damage to these neural cells could contribute to the acute respiratory issues seen in COVID-19 patients. Alternatively, with SARS-CoV it had been observed that coronaviruses can spread via synaptic transfer from chemoreceptors and mechanoreceptors within the lung to the medullary cardiorespiratory center [67].

### 3.2. Hyperinflammation

In addition to direct invasion, indirect effects induced by hyperinflammation and the “cytokine storm” also present a serious threat to neuronal cells. Data indicates COVID-19 can lead to AIDP, acute necrotizing hemorrhagic encephalopathy (ANHE), and stroke, amongst other neurological manifestations [46]. ANHE is a rare encephalopathy that occurs as a result of the blood-brain barrier breaking down in the presence of a cytokine storm, as can be ignited by viral infection [68]. Viral infiltration of the nervous system can trigger neuro-inflammatory responses resulting in microglia activation, which precipitates demyelinating processes; this is one of the primary etiologies for encephalopathy [69]. Even without direct virus infiltration, peripheral hypercytokinemia, which causes a neuro-inflammatory response that disrupts the blood-brain barrier and induces an imbalance of neurotransmitters within the central nervous system, has been identified as a factor in neuropsychiatric manifestations [70].

There are several routes through which SARS-CoV-2 can gain access to the brain. Additionally, indirect mechanisms of COVID-19 infection can impart equally as substantial damage on the nervous system. Aside from acute effects, it is of great clinical relevance to understand the lasting impact that COVID-19 infection may have on the brain.

### 3.3. Evidence of SARS-CoV-2 in Cerebrospinal Fluid (CSF)

There is ambivalence within the scientific community regarding the evidence of SARS-CoV-2 detection in cerebrospinal fluid (CSF) of patients. There are reports of singular case studies, by Oktar et al. [71] and Tee et al. [72], in which patients suffering from CNS symptoms underwent CSF analysis and SARS-CoV-2 was detected by PCR, a rebuke of these claims based on perceived limitations in study design [73], as well as several comprehensive reviews of CSF analyses in larger cohorts of patients diagnosed with SARS-CoV-2 [74,75,76]. Overall, there are instances in which SARS-CoV-2 is detected in CSF but it is a rare occurrence.

In a systematic review completed by Jarius et al. [73], 150 lumbar punctures performed on 127 patients that tested positive for SARS-CoV-2 via PCR and presented neurological symptoms across 17 European universities were retrospectively analyzed for trends in CSF. CSF PCR for SARS-CoV-2 was negative for 100% (76/76) samples analyzed. Additionally, a comprehensive case review was completed by Domingues et al. [74], analyzing 663 patients involved in 75 studies. Interestingly, 17% of patients experiencing encephalitis had detectable levels of the SARS-CoV-2 genome in CSF. In contrast, only 3% of patients experiencing COVID-19 encephalopathy had positive RT-PCR detection of SARS-CoV-2 in CSF. The authors attribute this discrepancy to lack of uniformity in case definition between studies.

Intermediately between the two frequencies of detection found by Domingues et al. [74], and Lewis et al. [75] calculated that 6% of patients diagnosed with COVID-19 and experiencing neurological symptoms. In a systematic review of 430 patients that tested positive for SARS-CoV-2 via PCR or serology, of the 303 patients whose CSF was assessed for presence of SARS-CoV-2 via PCR only 17 reported positive. Concordantly, in the same analysis only 6% of patients (14/252) who had direct or indirect evaluation for CSF SARS-CoV-2 antibodies presented evidence of intrathecal antibody synthesis [75]. While some studies are unable to detect SARS-CoV-2 in CSF, the studies that are able to detect its presence report its frequency at relatively low levels.

Distinct from the detection in CSF through PCR or antibodies, there are cases of virus, there are cases of virus being found in brain tissue from postmortem examinations of SARS-CoV-2 patients. Paniz-Mondolfi et al. [76] describe a case report in which a 74-year-old man with PD and COVID-19 was brought to the emergency room by his family after two falls at home. He continued to decompensate clinically and expired on day 11. Transmission electron microscope images of sections obtained at postmortem examination revealed the presence of 80 to 110 nm viral particles in frontal lobe brain sections [76]. Spherical viral-like particles were observed individually as well as in small vesicles of endothelial cells. Blebbing of viral-like particles coming in and out of the endothelial wall is presumed to be pathogen entry-transit across the brain microvascular endothelial cells into the neural niche. Neural cell bodies showed distended cytoplasmic vacuoles that contained enveloped viral particle exhibiting electron dense centers with distinct stalk-like projections.

Critically, the presence of SARS-CoV-2 was confirmed by testing frozen brain tissue and conducting four separate but parallel RT-PCR assays targeting distinct regions of the viral genome. SARS-CoV-2 was detected in the frozen brain tissue by all four RT-PCR assays [76].

Future studies have many questions left to investigate regarding the detection of SARS-CoV-2 in CSF, and the implications that this would have on direct CNS infection in COVID-19. Some of these questions include the timeframe, relative to infection and illness, in which SARS-CoV-2 is detectable in the CSF, what is the best detection method, is PCR sensitive enough to detect this virus, and how do we incorporate postmortem autopsy results into this framework?

### 3.4. In Vitro Evidence for an Interaction between SARS-CoV-2, α-Synuclein from Parkinson’s Disease, and Neuronal Cells

Although the link between other viral pandemics and neurodegenerative disorders is well-known [77], the relationship between SARS-CoV-2 and the brain remains under active investigation. Recently, the possible interaction of SARS-CoV-2 with elements of the brain that could promote PD was reported [78]. The hallmark of the neurodegenerative process in PD is represented by the intracellular presence of a protein named α-synuclein (αSYN), as reviewed by Lücking [79]. Furthermore, El-Agnaf and Irvine [80] summarized how αSYN accumulates in neuronal cells, leading to protein denaturation, nucleation, and aggregation. Early on, this protein aggregation spreads from neuron to neuron to disrupt dopaminergic transmission and function, eventually leading to neuronal death [79,80].

Semerdzhiev et al. [78] used an in vitro system to ask the question: could SARS-CoV-2 directly impact the PD process by interacting with αSYN? These researchers studied the ability of the two major SARS-CoV-2 proteins, specifically, spike protein (S-protein) and nucleocapsid protein (N-protein), on aggregation of αSYN directly and the impact of neuronal cell proteostasis [78]. They first measured the time it took for αSYN to aggregate in the absence of SARS-CoV-2 proteins. They found that the S-protein did not affect aggregation; however, the N-protein accelerated the αSYN aggregation rate almost 10-fold. Next, they micro-injected the S- and N-proteins into neuronal cells and found about twice as many cells died compared to the control. Furthermore, they noticed that the intracellular distribution pattern of αSYN was different in the cells treated with the SARS-CoV-2 proteins. While these are in vitro studies, they suggest a potential link between SARS-CoV-2 and αSYN to influence PD pathology [78].

### 3.5. Evidence That SARS-CoV-2 Infection Enhances Oxidative-Stress Induced Parkinsonism Models

**Figure 2:**
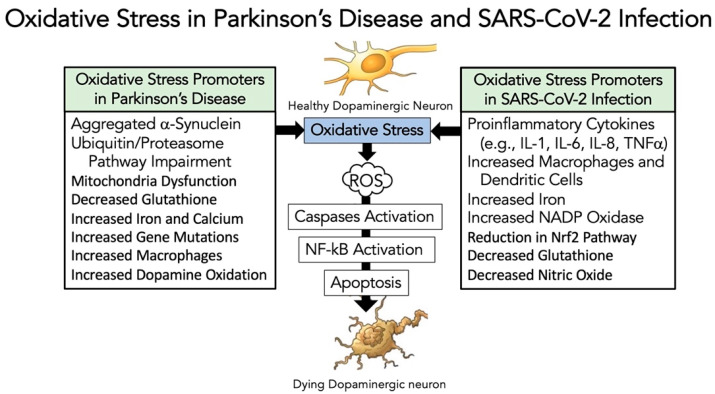
Overview of oxidative stress in PD and in SARS-CoV-2 infection. Abbreviations used: ROS, reactive oxidative species; NF-kB, nuclear factor kB; IL, interleukin; TNF, tumor necrosis factor; NAD, Nicotinamide adenine dinucleotide phosphate; Nrf2, nuclear factor-erythroid factor 2-related factor 2; ROS, reactive oxygen species.

As reviewed in Refs. [81,82,83], oxidative stress is a crucial element involved in both the cause and the progression of PD (Figure 1). Oxidative stress has also been linked to other processes involved in dopaminergic neuronal cell dysfunction, including mitochondrial malfunction, inflammation, excitotoxicity, and nitric oxide generation [81,82,83]. Oxidative stress generates reactive oxygen species [(ROS) including superoxide free radicals, hydroxyl radicals, and hydrogen peroxide] that promote cell damage leading to cell death. This oxidative damage leads to the induction of apoptosis through the activation of caspases [84]. Furthermore, ROS can have a stimulatory role in activating the critical NF-κB pathway [85]. Figure 2 highlights some of the promoters of oxidative stress in dopaminergic neurons in PD [81,82,83], and in SARS-CoV-2 infection [86,87,88,89].

If SARS-CoV-2 promotes or accelerates PD, one possibility is that it affects a similar pathway that follows in dopaminergic neuronal cell death. Chaudhry et al. [90] challenged dopamine-containing neurons comparing 6-hydroxydoamine (6OHDA)-induced cell death to SARS-CoV-2 infection. The results showed a similar stimulation of caspases occurring though the NF-κB pathway, with both test systems resulting in the death of the neuronal cells. In a different model, Musgrove et al. [91] found that oxidative stress with αSYN promoted a potent neuronal cell death. They showed using adenoviral constructs of αSYN and paraquat for generating ROS that oxidative stress promoted αSYN cell-to-cell transfer [91].

To further study this link between oxidative stress, PD, and SARS-CoV-2, Smeyne et al. [92] performed the following study: intranasal injection of virus into SARS-CoV-2-susceptible mice; a month later, SARS-CoV-2 and sham-treated mice were challenged with 1-methyl-4-phenyl-1,2,3,6-tetrahydropyridine (MPTP), which is a mitochondrial toxin that produces some of the characteristics of PD. The results showed no neuronal cell death from SARS-CoV-2 compared to saline controls. Furthermore, SARS-CoV-2 pre-treatment with MPTP led to substantially more neuronal cell death compared to control mice treated with MPTP. These results suggest that SARS-CoV-2 did not promote PD alone, but virus with mitochondria oxidative stress from MPTP resulted in more neuronal cell death compared to MPTP alone [92]. Additionally, mice treated with MPTP 45 days after infection with SARS-CoV-2 exhibited a 3-fold increase in activated microglia in the substantia nigra pars compacta region of the brain, significantly higher than uninfected mice treated with MPTP [92]. Overall, these results imply that SARS-CoV-2 enables the pathological changes found in PD (e.g., αSYN aggregation changes) versus simply causing dopaminergic cell death from virus alone.

Similarly, Fernández-Castañeda et al. [93] investigated SARS-CoV-2-mediated neuroinflammation, microglia activation, and the consequences of these processes on neurogenesis and cognitive function in a mouse model as well as human patients. They reported that following mild respiratory SARS-CoV-2 infection, in which no virus was detected in the brain by immunohistochemistry, white matter-selective microglia reactivity is present as determined by IBA1 and CD68 co-positivity via immunohistochemistry [93]. Reactive microglia have been shown to impair mechanisms of cellular homeostasis, myelin plasticity, and new neuron generation [94,95,96]. Furthermore, microglia cytokine signaling has been shown to induce neurotoxic astrocyte reactivity [97]. Mice exhibited elevated pro-inflammatory cytokines, such as CCL1, CXCL10, and CCL7, in CSF at least 7-weeks post-infection; CCL1 is associated with impairments in cognitive function [98]. Human patients experiencing “long-COVID” with cognitive symptoms (48 subjects) demonstrate similarly elevated plasma levels of CCL11 as compared to patients with “long-COVID” lacking cognitive symptoms [93].

Microglia cells and their mitochondria are critical participants in the innate immune response to infection, as reviewed by Tiku et al. [99], Ferger et al. [100], Khan et al. [101], and Harry et al. [102]. Mitochondria are involved in cellular homeostasis, but they also support the antiviral immune response by enabling the release of pro-inflammatory cytokines [99,100,101,102]. Singh et al. [103] reported that SARS-CoV-2 avoids the innate immune response by altering the function of mitochondria in microglia cells. Interestingly, the ACE2 receptor also regulates mitochondria function [104]. The binding of SARS-CoV-2 (S-protein) to ACE2 receptors on microglia cells reduces ACE2 receptor expression to decrease mitochondria energy (ATP) and activate NADPH oxidase, which generates ROS [103,105]. Thus, SARS-CoV-2 through ACE2 receptors on microglia cells could promote oxidative stress and ROS, and neuroinflammation leading to apoptosis in neighbor dopaminergic neuronal cells. Furthermore, Clough et al. [106] showed that SARS-CoV-2-infected microglia cells increased oxygen consumption, consistent with increased oxidative stress and ROS.

Additionally, Pliss et al. [107] found that SARS-CoV-2-induced ROS generation in microglia cells leads to damage to lipids and respiratory burst proteins, lowering mitochondria DNA levels. Therefore, one could envision a similar phenomenon occurring in SARS-CoV-2-mediated binding to dopaminergic neurons, expressing ACE2 receptors, as described by [108,109,110]. Finally, consider the consequences of SARS-CoV-2 promoting oxidative stress and ROS generation through mitochondria dysfunction in microglia and dopaminergic neuronal cells, which could promote neurodegeneration, as proposed by Di Filippo et at. [111]. The resultant neuronal disruption implies that SARS-CoV-2 infection could possibly alter the process of neurodegeneration, especially relevant to PD.

Overall, these in vivo results further support the in vitro studies illustrating that SARS-CoV-2 infection has profound and lasting impacts on several cell types in the brain that are implicated in the development of PD. Furthermore, these in vivo results potentially imply that a SARS-CoV-2 infection could worsen the clinical scenario in someone with pre-existing neurodegeneration. Another essential related feature is the increased risk of severe SARS-CoV-2 infection and developing PD linked to older adults. Thus, the next section involves an overview of the immune system and the impact of a reduced functioning immune system that typically occurs in older adults.

## 4. The Altered Immune System in Older Adults and Curious Roles for the Immune System in Parkinson’s Disease and COVID-19

### 4.1. Overview of the Immune System in Health and Disease

The immune system is a complex interacting network of organs and tissues, cells, and molecules designed to work together to identify and protect the body from infectious pathogens and other diseases, as presented by Abbas et al. in *Basic Immunology: Functions and Disorders of the Immune System* [112] and Murphy and Weaver in *Janeway’s Immunobiology* [113]. The immune system organization is based on cells and molecules with specialized roles in defending against infection with three defense mechanisms. The first line of defense against microorganisms is the intact skin and mucous membranes [112,113]. If microorganisms breach this line and enter the body, then the immune system’s innate (natural) arm (second line of defense) is available to destroy the invaders [112,113]. The first and second lines of defense cannot modify their response to pathogens. The third level of defense is specific protection provided by the immune system’s adaptive (acquired) arm; however, it takes several days for this defensive stage to fully function [112,113]. The two components of the adaptive arm are cell-mediated immunity and antibody-mediated (humoral) immunity. It is important to stress that all three levels of defense are critical for maintaining our health, and pathogenic substances have attempted to modify or mutate their structural components either to avoid or to evade our immune-based defenses.

### 4.2. Cytokines in Inflammation, Immunity and COVID-19 Infection

The immune system’s response to infection, injury, and toxic compounds is inflammation [112,113]. The inflammatory process is a host-defense response mechanism. Cytokines orchestrate many immune and inflammation reactions (for further review, see Refs. [114,115,116]). Cytokines are small soluble proteins produced in response to an antigen, and they serve as chemical messengers to control the innate and adaptive immune systems. They are produced by virtually all cells involved in innate and adaptive immunity. Furthermore, cytokines bind to specific cytokine receptors on other cells of the immune system and influence their activity in some manner. Importantly, uncontrolled inflammation can lead to host morbidity and mortality. In severe SARS-CoV-2 infection, there is potential for an exponential release of pro-inflammatory cytokines (termed Cytokine Storm Syndrome), resulting in uncontrolled inflammation and multi-organ damage with frequent progression to death (as described in Refs. [14,15]). This sudden and extreme hyperinflammatory response inappropriately leads to immune dysfunction and systemic inflammation that promotes and sustains multi-organ dysfunction and rapidly progresses to organ failure.

### 4.3. The Defective Immune System in Older Adults in the Absence and Presence of SARS-CoV-2 Infection

The biology of aging is complex; however, there is now substantial evidence that the immune system undergoes a series of changes linked to the aging adult [117,118,119]. The immune system’s lifelong engagement of pathogens in older adults results in a relatively common condition linked to age-associated frailty. This process has been named “inflammaging”, where systemically, one has increased baseline levels of inflammation, which results in the reduction of an immune response (reviewed in Refs. [117,118,119] and references cited therein). In the situation described for SARS-CoV-2 infection, where it can present with tremendous potential for a massive pro-inflammatory response, this result is frequently met with difficulty in the older individual. As summarized in Refs. [120,121,122], healthy but older individuals frequently have elevated C reactive protein levels and pro-inflammatory cytokines (particularly interleukin-6 and interleukin-8). Furthermore, they may be unable to clear dying or dead cells from the infected region of the body, a process termed senescence-associated secretory phenotype (SASP) [123]. Failure of both processes, an overabundance of inflammation and SASP, fails antigen-specific immunity, which could presumably reduce an older adult’s immune system’s response to infection.

The effect of SARS-CoV-2 infection is promoted by hyper-inflammation as described above. The result of hyper-inflammation leads to tissue damage, systemic cytokine storm, and thrombosis. Furthermore, there is supporting evidence that older adults have a less active type I interferon production and signal response [124,125], suboptimal T cell response due to a restricted T-cell receptor repertoire [126,127], decline in the humoral B-cell response [128], impaired antigen-presenting abilities of monocytes and dendritic cells [129], and hyporesponsive neutrophils [130], which would all contribute in a reduced response to severe SARS-CoV-2 infection. Thus, the collective dysfunction of the innate and adaptive immune systems in older adults could certainly contribute to increased susceptibility to COVID-19 when compared to younger age groups infected by SARS-CoV-2, as depicted by Akbar et al. [120], Bartleson et al. [121], and Odoj et al. [122].

### 4.4. Role of the Immune System in Parkinson’s Disease in the Absence and Presence of SARS-CoV-2 Infection

**Figure 3:**
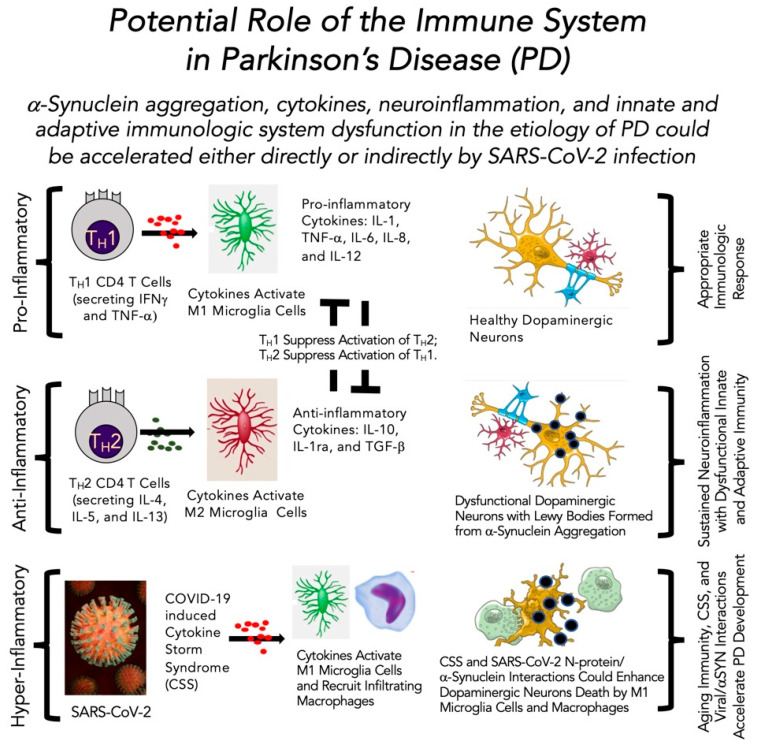
The role of the immune system in the development of PD. The **top-left** panel shows the adaptive immune system in promoting a pro-inflammatory state. The **middle-left** panel is the anti-inflammatory reaction to temper down the first reaction. The **bottom-left** panel shows the activation of adapting immune cells from SARS-CoV-2 hyper-inflammation. The **top-**, **middle-**, and **bottom-right** panels show the immunological consequences of the appropriate and inappropriate reactions of the immune system cells, respectively (dark-colored dots represent aggregated αSYN).

Several factors contribute to the development of PD (Figure 1). When added to advanced age as the leading risk factor for developing PD, the immune system’s prominent role, or lack thereof, becomes a factor in further understanding the etiology of the mid-brain degeneration of dopaminergic neurons seen in PD. The occurrence of intracellular inclusions named Lewy bodies is associated with the development of PD (for further review, see Refs. [79,80,131,132]). A major component of Lewy bodies is aggregates of the protein αSYN. Thus, aberrant aggregation of αSYN is likely a trigger for some or much of the biochemical changes within the dopaminergic neurons. A possible scenario leading to PD in older adults includes αSYN aggregation in dopaminergic neurons. The response to αSYN aggregation is cytokine-driven neuroinflammation, described by Odoj et al. [122], which enables an age-linked immunologic dysfunction as presented by Harms et al. [133] and Mayne et al. [134]. This pathophysiologic response leading to PD could potentially be accelerated either directly or indirectly by SARS-CoV-2 infection. There is also evidence that αSYN, normally an intracellular protein, becomes an autoantigen by being released into the extracellular neuronal cell spaces and then aggregates, which further activates the immune response to engage the now deranged neuronal cells [135,136,137]. Thus, over time, the afflicted neuron becomes dysfunctional and continues to be engaged by the inflammatory and immunological system cells and substances; dopaminergic neurons are destroyed and slowly, PD evolves over years (Figure 3).

Three steps are described in Figure 3 for the immune system’s potential role in the development of PD in the absence or presence of SARS-CoV-2. First, the pro-inflammatory state (neuroinflammation) prompted by the accumulating αSYN aggregates provide the first response (also see Refs. [138,139,140]) (upper left panel, Figure 3). The response has T_H_1 CD4^+^ T cells supporting a pro-inflammatory response to activate M1 microglia cells. Second, in a typical immunological process, the anti-inflammatory arm of the system turns to T_H_2 CD4^+^ T cells and their cytokines activate anti-inflammatory M2 microglia cells that down-regulate the pro-inflammatory process (middle left panel, Figure 3). This is the appropriate response to activate pro-inflammatory M1 microglia cells (the macrophages of the brain) that attempt to regulate the evolving problem, and then the appropriate anti-inflammatory response shuts it off (upper right panel, Figure 3), as described in [70]. Not shown here is the role of aggregated αSYN to induce innate immunity (as summarized in Refs. [140,141]). In addition, αSYN has been found to interact with Complement receptor (CR)3 and CR4, which potentially would promote phagocytosis [142]. Furthermore, there is an imbalance in PD to favor the pro-inflammatory CD4^+^ T cells, which would result in a sustained neuroinflammatory state, as recently described by Harms et al. [133] and Mayne et al. [134]. Combined with the known age-related dysfunction of the immune system, described above, neuroinflammation does not abate, further enhancing a detrimental immunological response in the mid-brains of unknowing PD patients (middle right panel, Figure 3). When an older adult with PD becomes infected with SARS-CoV-2, the tide turns even further to the neuroinflammatory pathway. Recall that an essential feature of COVID-19 infection is hyper-inflammatory, then either directly or indirectly, the ensuing cytokine storm syndrome provides further fuel to the immunologic fire already assembled in the mid-brain of someone with PD (lower right panel, Figure 3). This potential scenario of SARS-CoV-2 infection in an older adult with PD could co-exist alongside the altered immune system in the older adult to accelerate the disorder’s symptoms. We now enter a more clinically relevant section to describe the available evidence linking SARS-CoV-2 infection with either development of or progression of PD.

## 5. Evidence That COVID-19 May Promote, Support or Accelerate Parkinson’s Disease

### 5.1. Historical Perspective

The first connection between viral infection and subsequent development of parkinsonism was famously made by Constantin von Economo in 1917 [143]. Postencephalitic parkinsonism has been of interest since then, and with the global spread of COVID-19, of increasing concern. That concern has only been exacerbated by the growing body of literature highlighting both “long COVID” and “Neuro-COVID” [38]. SARS-CoV-2 is unusual in comparison to the original SARS virus in that it affects organs and systems beyond the lungs, including the brain [38]. Mounting evidence is drawing attention to the idea that neurobiological and psychobiological disturbances occurring during the acute phase of infection may persist well beyond recovery, with data showing neurological complications are not limited to severe cases or those with comorbidities, but rather extending to those with moderate symptoms and during recovery [38]. As mentioned by Makhoul and Jankovic [144], the link between Neuro-COVID and PD is supported by evidence dating back to 1992 showing that the basal ganglia can be infected by coronavirus in mice and that coronavirus antibodies can be isolated in the cerebrospinal fluid of patients with PD. Regarding the present threat of SARS-CoV-2, it appears that the virus both increases the vulnerability of those infected for PD and worsens PD symptoms, possibly even unmasking prodromal PD as some suspect regarding case reports about acute parkinsonism development.

### 5.2. Hypotheses

There are a variety of hypotheses exploring how infection with SARS-CoV-2 may predispose survivors to development of PD later in life. At the time of this review, those hypotheses with the most supportive data were protein-protein interactions, direct neural damage due to neurotropism, and inflammation.

Protein-protein interactions resulting in perturbations across networks is a significant cause of disease [145] and harkening back to von Economo, several RNA viruses have been associated with PD, including H1N1 [146], due to viral-driven host protein modification. RNA can be delivered to cells by exosomes, thus facilitating viral spread, neuropathogenesis, and immune evasion. Estrada [145] examined the 332 human proteins that COVID-19 interacts with and filtered them according to three criteria: direct interaction with SARS-CoV-2 proteins, significant expression in the lungs, and participation in human exosome formation. From that 24-protein subset, the author then identified those that interact with at least one of the 44 proteins reported to have significant association with PD and modeled a network demonstrating statistical significance that the two sets of proteins are a biologically connected cluster [145]. These data support SARS-CoV-2-perturbated proteins as a mechanism for PD as a sequela of COVID-19.

As previously mentioned, the ACE2 receptor is expressed throughout the brain, including both neurons and glial cells, as well as the endothelial cells lining the blood-brain barrier [147]. Possibly due to this compromise of the blood-brain barrier and supporting the neurotropism hypothesis, RNA from SARS-CoV-2 has been detected in the brains of some COVID-19 patients [148]. Due to the potential of neurotropism by the virus, it has been suggested that the neurological symptoms of infection may be due to neuroinvasion, neuroinflammation, and damage to the blood-brain barrier [38] rather than secondary to the respiratory manifestation of disease [143]. This suggestion is appropriate given that other coronaviruses exhibit a range of symptoms stemming from neuroinvasion and neurotoxicity, symptoms which often extend into post-recovery complications such as headaches, memory loss, and possibly neurological diseases including PD [38]. Of particular concern, however, is that SARS-CoV-2 exhibits the potential for PD-specific neurotropism as the ACE2 receptor is highly expressed in dopaminergic neurons [149].

### 5.3. Viral Invasion Linked Inflammation and Immunity

The connection between inflammation and the development of PD is well-established [150], with increased risk for PD exhibited by disorders of the immune system. Biomarker studies demonstrate continuous systemic inflammation in PD [148], which in the case of virally-induced inflammation, can affect the integrity of the blood-brain barrier [38]. This neuroinflammation is postulated to result in the death of dopaminergic neurons within the substantia nigra, thus inducing the paucity of dopaminergic neurons associated with PD [148]. Of interest to this particular point is SARS-CoV-2’s neurotropism directly affecting dopaminergic neurons [149]. As PD is associated with a decrease in dopamine and the neurons that produce it, the disease is additionally associated with an increase in the intraneuronal αSYN aggregates, forming Lewy bodies. Phillipens et al. [151] infected two species of macaques, a well-established animal model for COVID-19, with SARS-CoV-2 and was able to induce mild to moderate disease symptoms observed by both clinical signs and chest CTs. The macaques were euthanized five to six weeks after infection and their bodies underwent extensive postmortem and pathological investigation that was compared to healthy aged-matched controls of each species [151]. The authors found viral RNA in multiple areas of many of the monkeys’ brains and T cell-mediated inflammation in all of the brains, signifying the compromised integrity of the blood-brain barrier [151]. Most relevant, however, was the development of intracellular Lewy bodies in the caudate nucleus of five of the eight macaques in the experimental group [151]. Concerningly, these results mean that COVID-induced neuroinflammation is not limited to severe disease, but rather can develop even in the setting of mild or asymptomatic infection [151]. Supporting the evidence found in the macaques, a neuropathological postmortem study of 43 patients who died with COVID-19 found the same neuropathological signs of microglia activation and cytotoxic T cell activation in the brainstem, despite only three of those patients having PD [148]. There is an additional hypothesis that SARS-CoV-2 induces the type of initially neuroprotective upregulation in αSYN seen with the West Nile virus and Western Equine Encephalitis, which then risks prolonged elevated intraneuronal levels that lead to the formation of Lewy body aggregates [150]. Thus, inflammation from COVID-19 may promote the development of PD in a multifactorial fashion.

### 5.4. Neuropathological Alterations Caused by SARS-CoV-2

Given the concern for SARS-CoV-2′s impact on the brain, a number of studies have conducted postmortem examinations with particular focus on the brain, and two studies performed brain biopsies of patients who have passed away from COVID-19 [152]. The most common finding has been microgliosis and microglia activation, followed by hypoxic changes, and subsequently astrogliosis (as extensively described in Refs. [152,153,154,155,156,157,158,159,160,161,162,163,164,165,166,167,168,169,170,171,172,173,174] and summarized in [152]). These neuropathological findings are not unique to COVID-19, but rather have been found in the brains of patients infected by other viral encephalidities as well. Maiese et al. [152], Kantonen et al. [154], Matschke et al. [155], Thakur et al. [156], Poloni et al. [157], Jensen et al. [158], and Wierzba-Bobrowicz et al. [159] all posit that the neuropathologic changes they observed are the result of systemic inflammation caused by SARS-CoV-2, rather than by direct infection of neurons by the virus. This hypothesis is supported by data showing the virus is not reliably detected in the neuronal tissue of postmortem patients [175,176], and that the presence of SARS-CoV-2 is not associated with the severity of the neuropathological changes [155,175,176]. However, as noted in Section 3.3, Paniz-Mondolfi et al. [76], performed transmission electron microscopy of a postmortem brain and showed presence of viral particles in the frontal lobe. Additionally, Bulfamante et al. [176], compared the brains of two patients who passed away of COVID-19 to two controls and suggested that their results did not support hypoxemia as a cause for the COVID-19-related respiratory failure, but rather that SARS-CoV-2 directly targets the brainstem and the respiratory centers housed within it. They proposed that the difference in their results, compared to others such as Matschke et al. [155], was because they performed the examinations less than three hours after death, rather than between one and nine days, so that tissues would be maximally preserved [176]. Like Matschke et al. [155], however, they did detect both inflammation and SARS-CoV-2 in the brains of their patients [155,176]. Thus, it is still not entirely clear if the neuropathological changes seen in COVID-19 patients are caused by direct infection by the virus, or indirect effects stemming from systemic inflammation.

Interestingly, Colombo et al. [153], who performed postmortem examinations on 10 COVID-19 patients, found neurofilament scaffolding changes and axonal damage similar to that seen in neurodegenerative diseases, such as PD. Wierzba-Bobrowicz et al. [159], in their examinations of 52 COVID-19 patients, found accumulation of αSYN in two patients who developed parkinsonism. While no conclusions can be drawn from this data, these results are thought-provoking.

### 5.5. Existing Link with Neurological Consequences

Parkinsonism is a well-established repercussion of encephalitis produced by a number of viruses [146]. Data suggests that not only encephalitis, but perhaps even asymptomatic infection, may predispose survivors of COVID-19 to the development of PD later in life. Some even recommend monitoring individuals recovering from infection for neurodegenerative impairments, particularly for PD [38].

In addition to increasing vulnerability to PD, the COVID-19 pandemic has been shown to worsen symptoms of PD in those who were already known to have the disorder [150]. This worsening is felt to be multifactorial but includes contribution from increased psychological stress and time at home. Called the “hidden sorrow” of the pandemic, increased chronic stress is well-known to temporarily worsen the motor symptoms of PD [177]. The worsening of motor symptoms was highlighted strongly by Anghelescu et al.’s [178] qualitative study which involved interviewing patients with PD about the pandemic’s effects on their lived experiences. In relation, animal studies have demonstrated that sustained chronic stress may accelerate the rate of dopaminergic neuron loss in the context of an additional toxin [177]. Additionally, as seen in Antonini et al. [149] and Fasano et. al.’s [179] cases, PD patients with COVID-19 may require a prompt increase in dose of their dopamine agonists to counter motor decompensation during acute stress and fever that may result in severe generalized akinesia or akinetic crisis. It has been hypothesized that the stress of PD patients has been heightened further by the insufficiency of their ability to adapt as the acclimatization demanded by the pandemic necessitates normal dopaminergic functioning [177]. It is not uncommon for the dopamine paucity of PD patients to result in both cognitive and motor rigidity, and in the context of a pandemic such inflexibility can add to stress [177]. It is important to note that even prior to the pandemic, stress-related psychiatric symptoms were present in up to 30–40% of PD patients [177]. As stress related to the pandemic can worsen the motor symptoms of PD, so can the increased time at home. Homebound, PD patients may not only experience social isolation, but also be unable to go for a walk, attend a fitness class, or visit a physical therapist [177]. Given the significance of physical exercise in PD symptoms, this loss of exercise may aggravate motor symptoms, affect nonmotor symptoms such as constipation, and further exacerbate stress [177]. Thus, the circumstances of the pandemic are suspected to contribute significantly to the clinical worsening of PD symptoms.

While the impact of the pandemic mentioned above may be challenging to quantify, the possibility that COVID-19 may unmask prodromal PD has already been raised. Several case studies were published detailing patients with COVID-19 developing clinical parkinsonism within a few weeks of contracting the virus, are pooled by Brundin et al. [148] for discussion. Case reports by Faber et al. [143], Mendez-Guerrero et al. [180], and Cohen et al. [181] describe three patients who were 35, 45, and 58 years old; the two older patients required hospitalization due to the severity of their respiratory symptoms. The oldest of the three recovered spontaneously and at the time of the case study’s writing, was able to ambulate a few steps without support [180]. The other two patients were treated with a variety of dopaminergic drugs and experienced considerable improvement in their parkinsonism symptoms [143,148,181]. The 58-year-old underwent imaging that was significant for the DaT-SPECT demonstrating an asymmetric bilateral decrease in presynaptic dopamine uptake in the putamen [180]. The 45-year-old had imaging remarkable for decreased F-FDOPA uptake in the bilateral putamen on F-FDOPA PET scan [181]. The 35-year-old had imaging showing only decreased dopamine transporter density on the left putamen, despite bilateral clinical symptoms, a discrepancy which the authors attributed to “microstructural changes in other brain pathways” [143]. MRI was normal in all three patients, no viral RNA was detected in the CSF of both patients who were tested, and the one patient who was tested for genetic variants linked to PD was negative for them [143,148,180,181]. None of the patients had any family history of PD, nor did they endorse any prodromal symptoms of the disease, including constipation and REM sleep behavior disorder [143,148,180,181].

Furthermore, an additional study was published by Rao et al. [182] in 2022 detailing three more patients that developed parkinsonism while infected with COVID-19. These patients were all males aged 66, 72, and 74 years. The 66-year-old presented with hoarseness and cough that progressed to immobility, muteness, limb rigidity, and severe bradykinesia despite improvement in his vital and laboratory values [182]. MRI demonstrated bilateral temporal lobe gliosis, periventricular punctate white matter ischemic changes in bilateral frontal and parietal lobes, and age-related cerebral and cerebellar atrophy [182]. His parkinsonism symptoms improved with initiation and resolved with optimization of levodopa-carbidopa [182]. The 72-year-old developed orthostatic hypotension, loss of smell, cog-wheel rigidity, postural-instability, bradykinesia, freezing episodes, and falls beginning on day 5 of his COVID-19 hospitalization [182]. These symptoms did not resolve with his recovery from his respiratory illness, and only did after four months of levodopa therapy [182]. The 74-year-old patient developed rigidity, postural instability, motor slowing, and decreased mobility during his hospitalization for COVID-19 [182]. His MRI showed ischemic changes in the periventricular white matter [182]. His symptoms began to improve only with the initiation of levodopa-carbidopa therapy [182]. None of these three patients had any prodromal symptoms of PD prior to COVID-19 infection, and the improvement of their parkinsonism only with initiation of standard PD therapies futher supports a link between COVID-19 and the development of parkinsonism [182].

While these case reports do not prove a causal relationship between COVID-19 and the development of parkinsonism, the rapid onset of severe motor symptoms within weeks of infection is of interest to the scientific community as first noted by Faber et al. [143] in 2020 and reiterated again by Rao et al. [182] in 2022 as it remains unresolved. It has been posited that the development of parkinsonism after COVID-19 diagnosis may stem merely from prodromal parkinsonism becoming unmasked, though that was not true for the case studies presented by Brundin et al. [148], and is not known for those presented by Rao et al. [182]. The next section will expand upon this topic by examining the potential relationship between SARS-CoV-2 and atypical parkinsonism.

## 6. Evidence Linking SARS-CoV-2, Neuroinflammation, and Atypical Parkinsonism

### 6.1. Atypical Parkinsonism

Definitively distinguishing PD from other forms of parkinsonism is often difficult, with diagnostic criteria relying heavily on the rate and order of cardinal symptom development (rigidity, postural instability, bradykinesia, and resting tremor) together with the presence or absence of other numerous motor and non-motor symptoms, as reviewed in Refs. [28,183,184,185,186,187]. Multiple, primary parkinsonian syndromes (the so-called “atypical parkinsonisms”) have been recognized as pathologically-distinct, degenerative conditions which mimic several of the symptoms of PD, but patients with these alternate conditions find their disease progression to carry greater and earlier morbidity, alongside altered risks of mortality as compared to idiopathic PD (for additional information, please consult Refs. [28,183,184,185,186,187]). Further confusing the diagnostic landscape are the secondary forms of parkinsonism associated with extrinsic factors like heavy metal depositions (e.g., copper, manganese) or infectious viral agents like the 1918 pandemic influenza, prion-like diseases, and possibly now SARS-CoV-2 [188].

The atypical parkinsonisms include many conditions with some classic (but not necessarily common) presentations that can help to distinguish them clinically; but with sufficient overlap of symptoms that even seasoned movement disorder specialists can confuse [28,183,184,185,186,187]. Multiple system atrophy parkinsonian variant (MSA-P) can develop early dysautonomia and rapid use of a wheelchair for safety. Progressive supranuclear palsy (PSP) can develop early falls, swallowing difficulties, and extraocular muscle abnormalities in the form of a supranuclear gaze paresis or palsy. Corticobasal degeneration (CBD) can have a significant overlap with other degenerative diseases like Alzheimer’s, but diagnosis may be aided by a heavily asymmetric parkinsonian motor symptom alongside limb ataxia, apraxia, alien limb phenomenon, or hemibody sensory abnormalities. Dementia with Lewy Bodies (DLB) and frontotemporal dementia (FTD) present with cognitive difficulties within the first 1–3 years (if not earlier) of motor symptoms of parkinsonism. Much effort has been placed to similarly understand proposed pathophysiology of these conditions in tandem with research for PD; potentially offering insights into the role of neuroinflammation in primary degenerative conditions.

### 6.2. SARS-CoV-2 and Neuroinflammation in Atypical Parkinsonism

De Marcaida et al. [189] compared PD and atypical parkinsonism cases in their community setting with SARS-CoV-2 infection. The patient population at their Movement Disorder Clinic identified thirty-six patients (~61% idiopathic PD and ~20% atypical parkinsonism) who tested positive for COVID-19. Seventy-five percent of these patients presented with mental status alteration, and 42% had movement abnormalities, with a mortality rate of 36%. While this is a small data set, their research suggests patients with movement disorders (atypical parkinsonism or idiopathic PD) had an increased risk of death compared to control subjects. Cámara et al. [190] studied MSA patients exclusively during the COVID-19 pandemic. They followed 16 MSA patients and their caregivers that were under lockdown. The worsened symptoms were gait, speech, psychiatric, dysautonomic, dyskinesia, and sleep. The fatality rate was 25% (4 of 16), which should be noted that 6% of the MSA patients were positive for SARS-CoV-2. Although the study’s sample size was small, these results are still significant due to the rarity of the disease covered and the similarities of MSA to PD.

In 2007, Hawkes et al. [191] proposed that a combination of an infectious agent (likely a virus) and the response of the host defense inflammatory/immune system would promote neurodegeneration. This was termed the “dual hit” hypothesis for the development of idiopathic PD [191]. Atypical parkinsonism and secondary causes of parkinsonism linked to SARS-CoV-2 have recently been reviewed by Xing et al. [188]. Much of the information described earlier for SARS-CoV-2 being neurotropic, potential viral interaction with αSYN, and the role of neuroinflammation from microglia cells leading to neurodegeneration all support aspects of the dual hit hypothesis.

Neuroinflammation has been proposed to contribute to the pathogenesis of PSP [192], PSP-P [193], and MSA-P [194]. A key component is microglia cells promoting the release of pro-inflammatory cytokines and engaging/activating elements of both the innate and adaptive immune systems. A SARS-CoV-2 infection would further activate this mid-brain region while possibly engaging the neurodegenerative process linking microglia cell activation and chronic neuroinflammation as key participants in the pathogenesis of atypical parkinsonism [192,193,194].

The COVID-19 pandemic has resulted in many neuropsychiatric symptoms besides the expected pulmonary complications from SARS-CoV-2 infection. As with PD, the interaction between SARS-CoV-2 and atypical parkinsonism is not confirmed. However, what is becoming more evident is the reaction of microglia cells and T-cells to promote a potent neuroinflammatory response to an infectious substance like SARS-CoV-2. The neuroinflammation could possibly promote neurodegeneration that leads to atypical parkinsonism or PD [195,196]. Therefore, in the next section, we switch things around and ask if PD has a negative impact or modifies or worsens the symptoms or outcome of SARS-CoV-2 infection?

## 7. Evidence That PD May Worsen SARS-CoV-2 Infection Symptoms and Outcomes

It is worth noting first that the infection with SARS-CoV-2 has been shown to worsen PD symptoms dramatically [150,197]. The systematic review by Jaiswal et al. [197] pooled data from 16 studies for a total of 1290 patients with PD who tested positive for COVID-19 and demonstrated worsening of motor symptoms for between 19% to 100% of patients depending on the study. Summarizing the published cases, the review indicates that PD patients experience a worsening of their disease and a high risk for mortality, ranging between 5.7% and 100% [197]. This high risk for mortality can be linked to the hypothesis that a prior diagnosis of PD may worsen both the symptoms and outcomes of SARS-CoV-2 infection (reviewed in Refs. [146,149,198]). While Salari et al. [198] concluded that there was not a significant link between COVID-19 infection and the development of motor symptoms, the authors did note that PD symptoms overall during the pandemic did worsen for the reasons addressed above.

The hypothesis that PD may worsen SARS-CoV-2 infection has intrigued many, with multiple studies published recently investigating the idea (for further information, please see Refs. [147,149,150,177,179,199,200]). Symptoms of PD suspected to contribute to a more severe COVID-19 experience and outcome include respiratory muscle rigidity, poor cough reflex, and abnormal posture (reviewed further in Refs. [97,179,199,200]). Likely associated with the increased aspiration risk of PD patients, pneumonia has been noted as the most common cause of both inpatient admissions and death for patients with PD [147,177,179,200]. The cross-sectional study conducted by Scherbaum et al. [201] found that PD patients are more likely to have the comorbidities linked to more severe COVID-19 infection, and that the mortality rate for patients with PD diagnosed with COVID-19 are much higher than the general population, at 35.4% versus 20.7% overall. The increased mortality risk for PD patients was additionally bolstered by the results of Artusi et al.’s systematic reviews [202,203]. However, some studies argue that mortality data is inconclusive [150,204]. Multiple studies do note, though, that patients with longer histories and more severe PD have worse outcomes than more newly diagnosed, less severe PD patients (for additional information, please consult Refs. [149,150,204]).

## 8. Does SARS-CoV-2 Modify Neurodegenerative Processes in Parkinson’s Disease?

### 8.1. Theories That SARS-CoV-2 Promotes the Development of Parkinson’s Disease: Pros and Cons

In Table 1, several studies support SARS-CoV-2 as a potential cause of PD, and one study supports the concept of the virus as an unmasking property of prodromal PD. Also shown in Table 1 is one study showing no detection of SARS-CoV-2 in the CSF and one study that suggests αSYN is upregulated as an anti-viral host defense response that could, over time, promote the development of PD. Although still not definitive proof, numerous studies imply the possibility of a sinister role for SARS-CoV-2 with the brain, which possibly leads to neurodegeneration and onward in the susceptible person to develop PD.

### 8.2. Potential Pathways of SARS-CoV-2 to Modify Neurodegeneration

**Figure 4:**
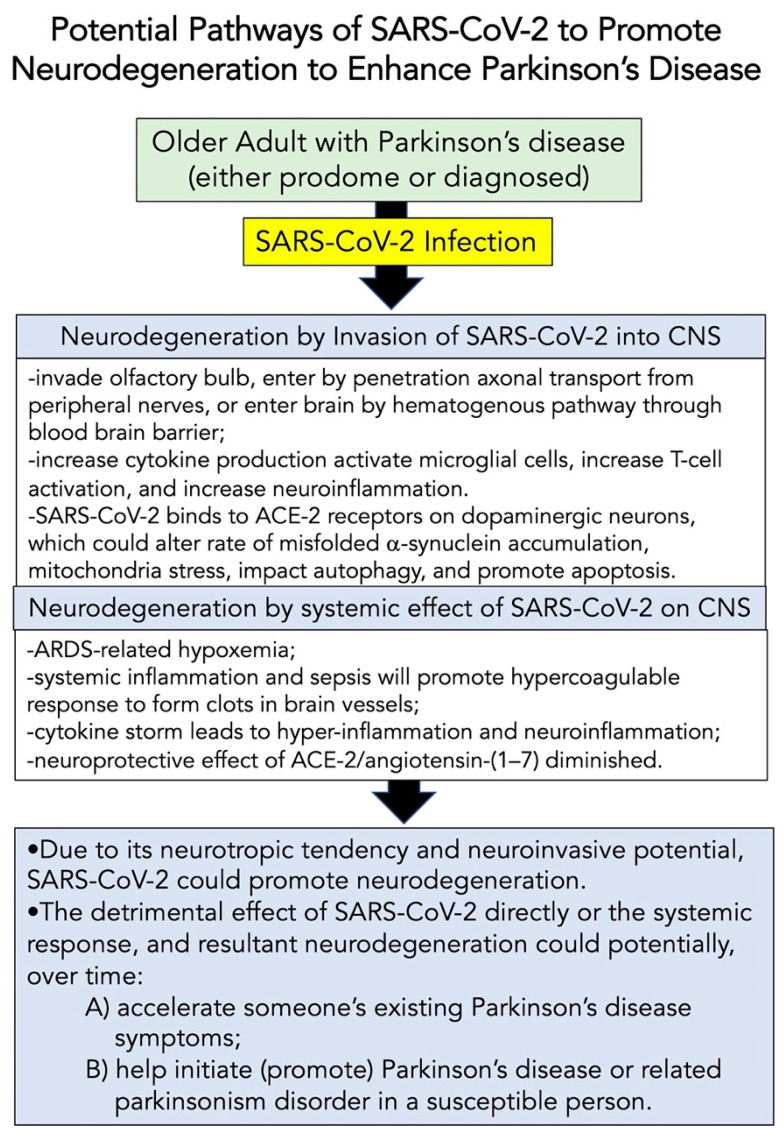
Potential pathways for SARS-CoV-2 to promote neurodegeneration, Parkinson’s disease, or related disorders.

Similar to studies already described above, Leta et al. [209] studied a group of 27 PD patients who had the “long-form” of COVID-19 infection; following the viral infection, where ~52% showed worsening of motor function, 48% needed increased levels of levodopa, and 22% had “brain fog” and other cognitive defects. An important question is whether these changes in PD symptoms were a direct consequence of SARS-CoV-2 infection? Or were these subacute changes similar to that studied by Zheng et al. [210] that have been noted in the past for PD patients having their symptoms made worse by infection? Notably, the current concern is, does SARS-CoV-2 increase the possibility of developing PD years from now, and does it genuinely participate in the progression of present PD symptoms? There is growing evidence to support the neurotropic potential of SARS-CoV-2 (as reviewed in these Refs. [9,10,11,12,13,63,211,212]). Figure 4 summarizes the possible neuroinvasive pathways that could be taken by SARS-CoV-2 to potentially promote neurodegeneration, which could accelerate existing PD or support the disorder’s development.

### 8.3. Future Directions

Several scenarios support the notion that SARS-CoV-2 is neurotropic. However, it is still unclear if the virus promotes a neurodegenerative process. Affirming and documenting the pathway(s) taken by SAR-CoV-2 would provide substantial validity to the virus promoting neurodegeneration. Likewise, carefully monitoring the recovery of confirmed SARS-CoV-2 cases in PwP (along with the COVID-19 vaccination status) would provide much information about whether SARS-CoV-2 infection exacerbates this neurological condition. Finally, the results described here and in many other outstanding reviews partly but not entirely support a causal relationship between SARS-CoV-2 infection and developing PD. An international effort is needed to verify or refute this relationship of SARS-CoV-2 with PD.

### 8.4. Limitations

There are several limitations to this review. First, the statistics are lacking worldwide to describe how many older adults with PD also have been infected by SARS-CoV-2. Second, only time will tell if older adult individuals with milder COVID-19 symptoms are spared from the patients described to date with a PD-like syndrome. Third, the impact of vaccination against SARS-CoV-2 and its effect on the various neurological complications have not yet been reported. Finally, as with other viral pandemics, we will only know if 20–30 years from now there is a spike in PD throughout the world due to the chronic neuroinflammatory state established in that patient following COVID-19 illness at a younger age.

## 9. Conclusions

Though much remains to be investigated regarding the effects of COVID-19 on PD, evidence suggests that some connection between the two diseases exists. In a little over a year, data has arisen worldwide that SARS-CoV-2 may increase the propensity of survivors to develop PD later in life and for those already diagnosed with PD, negative sequelae as a result of the pandemic from a variety of sources. These added factors only add to the universal increasing risk that is most important: naturally aging [148]. Thus, as more time passes, we may see an increase not only in acute COVID-19-induced parkinsonism, but also diagnoses of PD initially triggered by infection with SARS-CoV-2 decades prior.

## Figures and Tables

**Table 1:** Hypotheses and Studies that SARS-CoV-2 Promotes the Development of Parkinson’s Disease.

Study Supports: Cause/Unmasking/Not Support	In Vitro/In Vivo/Clinical	Primary Conclusions
Cause	Clinical	Three published single-case reports describing patients with COVID-19 developing clinical parkinsonism within 2–5 weeks of contracting SARS-CoV-2. As seen with West Nile virus and Western Equine Encephalitis virus, α-synuclein expression increases during viral infection of CNS, suggesting that SARS-CoV-2 infection could predispose individuals to the development of PD later in life. Brundin et al. [148]
Cause	Clinical	A case study of an elderly diabetic woman with parkinsonism with akinetic mutism following non-dyselectrolytemic osmotic demyelination syndrome, which was precipitated by COVID-19 infection induced hyperglycemic hyper-osmolar state. She was placed on levodopa/carbidopa and pramipexole, and after two months of follow-up her features of parkinsonism improved significantly, but with only mild improvement of the features associated with akinetic mutism. Ghosh et al. [205]
Cause	Clinical	A previously healthy 31-year-old man tested positive for COVID-19 and developed acute necrotizing encephalopathy (ANEC), with presenting features of parkinsonism and myorhythmia. Myorhythmia can also occur alongside other movement disorders, such as dystonia and parkinsonism, due to disrupted basal gangliathalamo-frontal cortical circuits. The exact pathogenesis of ANEC is not entirely clear, but systemic inflammatory insult and hypercytokinemia have been postulated to trigger necrotic brain lesions in patients with ANEC. Ong et al. [206]
Cause	In vitro	Identification that the SARS-CoV-2 nucleocapsid protein (N-protein) induces the aggregation of αSYN in a test tube. In the presence of N-protein, the onset of α-synuclein aggregation into amyloid fibrils is strongly accelerated, indicating that N-protein facilitates the formation of a critical nucleus for aggregation. These experiments suggest that SARS-CoV-2 infection and PD might originate from a molecular interaction between virus protein and α-synuclein. Semerdzhiev et al. [78]
Cause	Clinical	Three patients developed parkinsonism while infected with COVID-19, all of whom required levodopa-carbidopa therapy for recovery despite having no prodromal PD symptoms prior to COVID-19 infection. The authors concluded that parkinsonism could be a post-COVID-19 sequelae. Rao et al. [182]
Cause	In vivo	Authors infected macaques with SARS-CoV-2 and demonstrated brain inflammation and post-mortem studies uncovered Lewy bodies were not present in controls. They conclude that this data is a serious warning for potential COVID-19-related neurodegeneration (particularly PD given the presence of hallmark Lewy bodies) even after asymptomatic or mild infection. Philippens et al. [151]
Cause	Clinical	Two cases of patients with COVID-19 encephalopathy who developed parkinsonism without a history of prodromal PD symptoms. FDG-PT/CT imaging showed distinct areas of hypo- and hyper-metabolism in comparison to 48 healthy controls. Authors state that while they cannot dismiss symptom development due to unmasking, given the patients’ lack of prior PD prodromal symptoms and motor features prior to infection, rapid onset of parkinsonism after encephalitis, and lack of improvement after discontinuing neuroleptics and initiating levodopa, that is unlikely. Morassi et al. [207]
Unmasking	Clinical	Single case report of patient who developed parkinsonism within days of COVID-19 symptom onset. Makhoul and Jankovic [144]
Not Support	Clinical	CSF PCR for SARS-CoV-2 was negative for 100% (76/76) in samples analyzed that were assessed previously and were positive for SARS-CoV-2. Jarius et al. [73]
Not Support/Cause	In vivo	RNA viruses upregulate αSYN in neurons, which subsequently can activate the interferon-mediated-anti-viral defense mechanism in innate immunity. However, long term consequences could lead to chronic inflammation with the development or progression of PD. Rosen et al. [208]

## Data Availability

Not applicable.
